# Mental Health of Adults Treated in Adolescence with Scoliosis-Specific Exercise Program or Observed for Idiopathic Scoliosis

**DOI:** 10.1155/2014/932827

**Published:** 2014-01-20

**Authors:** Maciej Płaszewski, Igor Cieśliński, Roman Nowobilski, Tomasz Kotwicki, Jacek Terech, Mariusz Furgał

**Affiliations:** ^1^Faculty of Physical Education in Biała Podlaska, Institute of Physiotherapy, Warsaw University School of Physical Education, Akademicka 2, 21-500 Biała Podlaska, Poland; ^2^Faculty of Health Sciences, Institute of Physiotherapy, Jagiellonian University, Michałowskiego 12, 31-126 Kraków, Poland; ^3^Department of Pediatric Orthopedics and Traumatology, University of Medical Sciences of Poznan, 28 Czerwca 1956r. No. 135/147, 61-545 Poznań, Poland; ^4^Centre of Pulmonology and Thoracic Surgery, J. Fałata 2, 43-360 Bystra, Poland; ^5^Department of Psychiatry, Jagiellonian University Medical College, Kopernika 21A, 31-501 Kraków, Poland

## Abstract

*Objective*. To examine general mental health in adult males and females, who in adolescence participated in a scoliosis-specific therapeutic exercise program or were under observation due to diagnosis of scoliosis. *Design*. Registry-based, cross-sectional study with retrospective data collection. *Methods*. Sixty-eight subjects (43 women) aged 30.10 (25–39) years, with mild or moderate scoliosis (11–36° Cobb angle), and 76 (38 women) nonscoliotic subjects, aged 30.11 (24–38) years, participated. The time period since the end of the exercise or observation regimes was 16.5 (12-26) years. Beck Depression Inventory (BDI) and General Health Questionnaire (GHQ-28) scores were analyzed with the *χ* ^2^ and *U* tests. Multiple regression analyses for confounders were also performed. *Results*. Intergroup differences of demographic characteristics were nonsignificant. Scoliosis, gender, participation in the exercise program, employment, and marital status were associated with BDI scores. The presence of scoliosis and participation in the exercise program manifested association with the symptoms. Higher GHQ-28 “somatic symptoms” subscale scores interacted with the education level. *Conclusions*. Our findings correspond to the reports of a negative impact of the diagnosis of scoliosis and treatment on mental health. The decision to introduce a therapeutic program in children with mild deformities should be made with judgment of potential benefits, risks, and harm.

## 1. Introduction

Scoliosis, in its most frequent adolescent idiopathic form diagnosed in about 80% of the cases [1, 2], is the most prevalent orthopedic condition affecting children [2] and may have lasting consequences [3, 4]. Adult deformities can be classified as progressive adolescent idiopathic, primary degenerative, *de novo*, or secondary degenerative scoliosis [1]. In the adult population, the prevalence of scoliosis may exceed 30% [1, 3]. It ranges from nearly 9% in 40-year-olds to 68% in 70-year-olds [5] and increases almost linearly from the 6th to the 8th decade of life [6]. In contrast to adolescents, no associations with gender have been observed in adults [1–3].

Spine deformity may significantly influence the general health and patient's self-reported health-related quality of life (HRQoL), with a poor correlation between the radiographic and clinical findings [2, 3, 7]. Limitations in participation in social life and intimate relationships, lower marriage rates, fear of injury, poor self-perception, difficulties on the labor market, and mental disorders have been reported [1–3, 7]. Surgery or brace treatment may also lead to psychological side effects [3, 4, 8, 9]. Long-term outcome reports [10], including the Iowa [11], Montreal [12], and Göteborg [4, 13] series, suggested that deformity causes psychopathological effects or demonstrated positive coping mechanisms [3, 12]. Furthermore, the available papers focus on the assessment of health-related quality of life and utilize generic (typically the SF-36 or WHOQOLBREF questionnaires) or condition-specific (e.g., the Scoliosis Research Society (SRS)-22 questionnaire) measures with their physical, social, and emotional roles, mental functioning, bodily pain, and body image components, incorporated to produce a measure of a person's perceived general health status [5, 7]. Moreover, there are only few short-term reports comparing men and women [10, 14]. Also, we found no reports on scoliosis-specific exercises in the context of mental health and well-being, although physical exercises in the treatment of scoliosis remain a matter of debate [15] and high-level evidence for or against these procedures is lacking [16].

To our best knowledge, there has been no investigation utilizing the Beck Depression Inventory (BDI) and the Goldberg General Health Questionnaire (GHQ) in patients with scoliosis, despite the fact that these instruments are highly popular and widely used in primary health care settings and general populations [17].

Therefore, the aim of our study was to examine general mental health in young adult men and women, who in adolescence participated in a specific therapeutic exercise program or were under observation due to diagnosis of idiopathic scoliosis. We tested the null hypothesis that the participation in scoliosis-specific exercise regime in adolescence is not associated with mental health and well-being in adult life.

To improve the quality of reporting, we followed the recommendations of Strengthening the Reporting of Observational Studies in Epidemiology (STROBE) statement [18].

## 2. Methods

### 2.1. Study Design and Participants

We recruited participants by a cross-sectional evaluation from the population of subjects examined for scoliosis at the centre of Corrective and Compensatory Gymnastics, Bielsko-Biala, Poland. The Centre provides scoliosis screening for schoolchildren from the urban and suburban population of about 300 000. We analyzed the medical records of 5017 children examined between 1984 and 1995. We considered eligible registries of those children in whom observation or exercise program were recommended, while bracing and/or surgical treatment was exclusion criteria. Because of financial and technical limitations, we based our study on a sample of subjects. For that reason, we subsequently randomly selected 250 registries. We performed a simple random sampling and used a random numbers table for that purpose.

**Figure 1:**
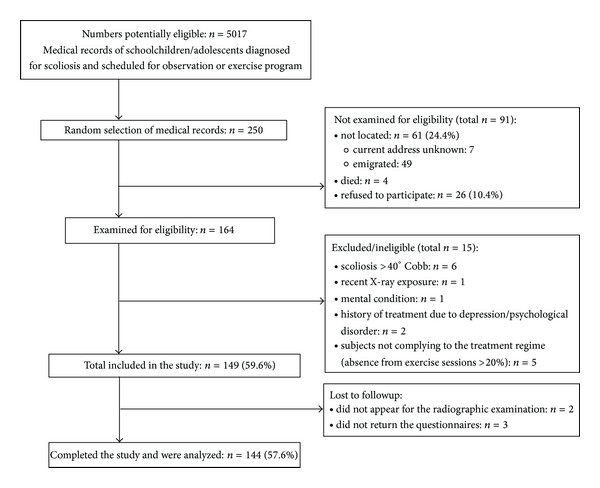
The process of recruiting participants.

The largest of the previous observational studies in adults, who in adolescence were braced or treated surgically or observed, included 1476 patients and 1755 controls [12], while other major studies included 145 surgically treated, 127 braced, and 100 control subjects [4] and 40 observed and 37 braced patients [13]. Response rates in those, and other studies, described by Goldberg et al. [12], ranged from 48% to 89%. Therefore, based on our pilot study, we presumed to enroll at least 60% of potential participants. Comparing to the analyzed studies we assumed that this sample size was sufficient for the study with a margin of error of 3% and a 95% confidence level, based on an actual estimate of 50%. We made every effort to locate the subjects and, if they changed their addresses, we attempted to retrieve the current addresses or telephone numbers from their parents or other inhabitants or collected further relevant information, for example, about migration. We applied the procedures recommended to increase participation (we published an invitation letter in a local free newspaper, provided personalized introductory letters, and made follow-up telephone calls to nonrespondents) [19]. We managed to locate 164 potential participants, of whom 15 were ineligible due to severe scoliosis (*n* = 6), recent X-ray exposure (*n* = 1), mental condition (*n* = 1), history of treatment of depression or other psychological disorders (*n* = 2). Five people were excluded due to noncompliance with treatment regimen (the rate of absence from exercise sessions exceeding 20%, based on patients' records). Of 149 participants finally included in the study, 2 dropped out and 3 did not return the questionnaires. Thus, a total of 144 (57.6%) of the initially selected subjects completed the study. The flow of recruitment and the selection criteria are presented in detail in Figure 1.

Mean age at diagnosis was 10.5 (range 9–16) years for the whole group, and treatment started for subjects aged 10.7 (9–16) years. The regime involved scoliosis-specific, symmetrical, strengthening, antigravity, and elongating exercises of the postural muscles, performed in group during 45-minute gym sessions twice a week and individually at home (sets of 12–15 exercises, 30–45 minutes a day). Subjects who did not participate in the exercise program were observed for three to five years on the basis of scheduled follow-up orthopedic examinations. Follow-up period since the termination of treatment was 16.5 (12–26) years for the whole group, 17.1 (12–25) years for the exercising group, and 15.9 (12–23) years for the observed subjects. The differences in the distribution of variables were nonsignificant (Table 1).

### 2.2. Curve Measurements

Two specialists independently measured the magnitude of the curvature according to the Cobb method [20], on a full-length anteroposterior radiograph, and then a consensus was reached. On this basis, we divided participants into two groups: persons with mild (11–24° Cobb) or moderate (25–44° Cobb) scoliosis and nonscoliotic participants (Tables 1 and 2).

### 2.3. Outcome Measures

The BDI, in its long form, is a 21-item self-reported measure, with items rated 0 to 3 (most severe signs and symptoms) [17]. We applied the BDI-I in its Polish adaptation [21] and interpreted the results according to the classification proposed for this version [22]: 0–4 no or minimal, 5–13 mild/low, 14–20 moderate/medium, and ≥21 severe depression. We additionally analyzed the results with respect to the proposed 10-point threshold for depression [23]. Subjects responded on a seven-day recall basis, so that we obtained results reflecting a trait rather than a state. Only the total score can typically be interpreted. However, because interesting single-symptom comparisons had been conducted [24], we also performed such analyses.

The assessment of depression alone can be oversimplified, as many depressive symptoms can be the expression of the condition itself [25]. Thus, we decided to use Beck's BDI for the assessment of depressive mood experienced in a recent period of time, because it contains (unlike other scales) relatively few symptoms that can be ascribed to the somatic state of patients. For the assessment of potential psychiatric morbidity of a wider spectrum, we applied the GHQ-28. Apart from depression, the tool assesses somatisation, hypochondriasis, anxiety, sleep, and social functioning disorders.

The GHQs are self-administered instruments designed to detect a probable psychiatric disorder. Different versions have been developed, with the most widely used 28-item GHQ-28 [17, 26], validated into the Polish context, and available in Polish version, published in Poland [27]. The GHQ-28 consists of four subscales: “somatic symptoms,” “anxiety and insomnia,” “social dysfunction,” and “severe depression” [26, 27]. The results are interpreted within a given group and in accordance with the maximum possible scores (21 in domains and 84 general total in the Likert scale).

### 2.4. Statistics

We used descriptive statistics for the demographic and clinical characteristics of the subjects. The maximum-likelihood chi-square test was used to assess intergroup differences for subsequent characteristics and individual BDI symptoms, while the Mann-Whitney *U* test was used for categorical data (BDI and GHQ-28 total and GHQ-28 domains). To examine the interaction between the BDI scores (as categorical data) and a number of confounders (Table 5), we employed the count data regression model zeroinfl( )/hurdle( ), from the PSCL package [28, 29]. We chose that model because the total BDI scores were dispersed, the number of zero scores was considerably large, and, additionally, the distribution of the remaining scores (after the exclusion of zero scores from the analysis) was close to the Poisson distribution. The GHQ-28 scores were free from such constraints; therefore, we used the polr( ) function of the MASS package—the logit model for ordinal variables [30].

## 3. Results

**Figure 2:**
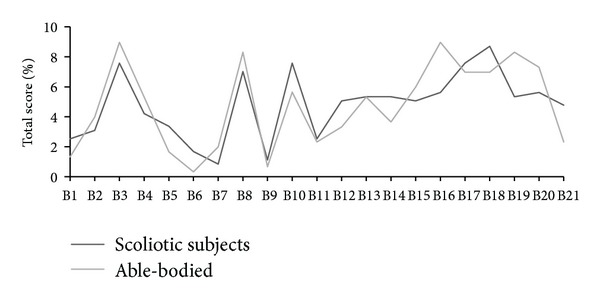
Scores of individual BDI symptoms in groups. BDI items: B1: mood; B2: pessimism; B3: sense of failure; B4: lack of satisfaction; B5: guilt feelings; B6: sense of punishment; B7: self-dislike; B8: self-accusation; B9: suicidal wishes; B10: crying; B11: irritability; B12: social withdrawal; B13: indecisiveness; B14: distortion of body image; B15: work inhibition; B16: sleep disturbance; B17: fatigability; B18: loss of appetite; B19: weight loss; B20: somatic preoccupation; B21: loss of libido.

Median total BDI scores of both scoliotic and nonscoliotic groups (4.60 and 5.89, resp., *P* = .77) were within the category of mild depression [22]. Both groups differed significantly in the distribution into the categories of minimal, mild, moderate, and severe depression (*P* < .01), with more scoliotic than nonscoliotic participants showing depressive symptoms (45% and 33% of subjects, resp.). The differences between the subjects with mild and moderate deformities were also significant (*P* < .05), with a greater tendency for depressive symptoms in subjects with milder deformities. Only 11 (14%) nonscoliotic and 7 (10%) scoliotic participants exceeded the 10-point threshold. The results are shown in Table 3, and Figure 2 illustrates individual BDI symptoms.

**Figure 3:**
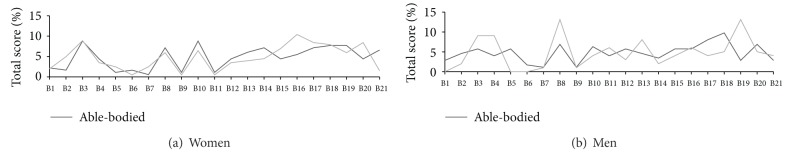
Scores of individual BDI symptoms—scoliotic and unaffected women (a) and men (b). Individual symptoms (B1-B2) are labelled in Figure 2.

Intragroup gender differences were nonsignificant for both the total BDI score and the categories of depression severity. Nonetheless, more women than men exceeded 10 points, irrespective of the group (Table 4). Figures 3(a) and 3(b) illustrate additional comparisons of the individual symptoms between men and women.

**Figure 4:**
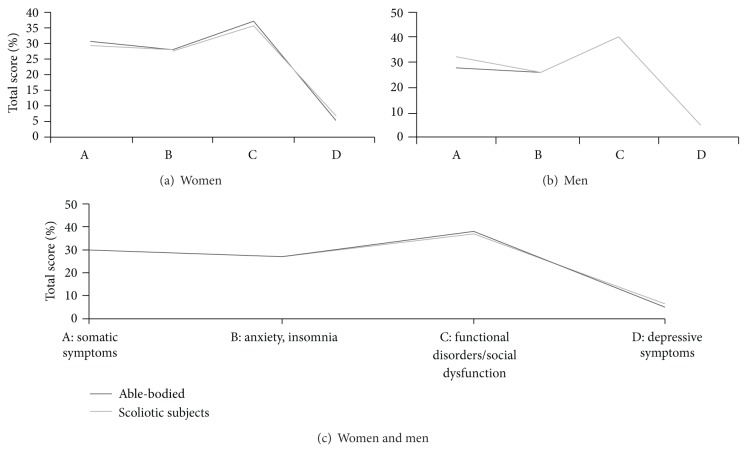
GHQ-28 subscales—scoliotic versus unaffected women (a), men (b), and women and men (c).

We obtained low scores of the GHQ-28 domains, with the highest scores of “anxiety/insomnia” both in scoliotic (3.99, 19% maximum) and in unaffected individuals (3.46, 16.5% maximum). Differences between both groups and between subjects with mild and moderate scoliosis were nonsignificant (Table 3 and Figure 4(c)).

Table 4 and Figure 4 demonstrate intergroup comparisons of the GHQ-28 subscales. The differences were significant only in the “somatic symptoms” subscale for intragroup gender comparisons in the nonscoliotic group, with lower scores in men (*P* < .05). In all comparisons, the overall mean and median values were far below the maximum possible results. None of the scores exceeded the figures of 11 for the subscales, which may be considered as achieving “caseness” [26].

For the multiple regression analysis for both BDI and GHQ-28—with the total score and subsequent domains analyzed—we included age, gender, presence of scoliosis (>10° Cobb), treatment type (exercise regime versus observation), marital status, employment, level of education, and presence of scoliosis in relatives as potential confounders. We only report findings with significant or nearly significant differences (Table 5). Therefore, for the GHQ-28, only domain A (somatic symptoms) is presented.

Gender, employment, and marital status were associated with BDI results (Table 5). Being male (.83 odds ratio, OR, .71–.98 confidence interval, CI), single (.82 OR, .68–.96 CI), employed (.80 OR, .54–.92 CI), and having lower level of education (.70 OR, .62–.79 CI) seemed to decrease the tendency for depression, while the presence of scoliosis (.84 OR, .72–1.00 CI) and participation in the exercise program (1.16 OR, .90–1.38 CI) manifested association with the symptoms. The difference was not significant (*P* = .06), but taking into account a considerably low power of the study (0.16 for BDI and 0.10 for GHQ-28, a posteriori) and the risk for type II error, we cautiously considered these findings as indicating a possible tendency.

We found a significant negative interaction (*P* < .05) between the level of education and somatic symptoms in GHQ-28 subscale A (.52 OR, .33–.85 CI). Also, being male seemed to be negatively associated with the tendency towards somatization (*P* = 1.87, where *P* = 2.00 is the minimum level of significance). In general, with lesser confidence (nonsignificant associations but with high odds ratios), the multiple regression for the GHQ-28 scores confirms the BDI findings.

## 4. Discussion

There are no set thresholds for the interpretation of the GHQ-28 scores, but nonetheless both the total and domain scores in the scoliotic and nonscoliotic groups were below the 11-point threshold of possible “caseness” [26]. Furthermore, the intragroup comparisons revealed that the GHQ-28 scores did not differ significantly between subjects with mild and moderate spine deformities (Table 3). The more specified analysis with the BDI confirmed that scoliotic subjects manifested more apparent depressive symptoms, irrespective of the severity of the deformity (Table 4). Considering that mild deformities are typically unnoticeable, it seems that it is the awareness of the deformity and not the physical manifestation itself that may play a role here. The multiple regression analyses (the zero inflation model for BDI and the logit model for ordinal variables for GHQ-28) confirmed these observations (Table 5). We found a significant association between depressive symptoms and the presence of spine deformity (*P* < .05). Depression can be an understandable response to loss and injuries to self-esteem associated with the diagnosis established in adolescence, which is the most vulnerable period for the formation of adult identity [25, 31]. The diagnostic label itself can have a significant influence on the patient's behaviour and emotions, even if there are no symptoms or diseases. Other factors for an increased risk of emotional disorders can be social stigmatization and exclusion associated with participation in a rehabilitation program [31].

There are no results available from similar studies regarding adults with a history of participation in scoliosis-specific exercise programs, but studies on adolescent patients treated with a brace indicate that scoliosis and therapy are significant risk factors for depression, suicidal thoughts, worry and concern over body development and appearance, and peer reactions, not only in girls, but also in boys [32]. Outcome studies on adults are less consistent. Nonsignificant effects on emotional reactions and mental health have been observed [3, 4, 8], and patients did not experience psychological distress or presented good coping mechanisms [12, 32]. The scores of the mental health component of the SF-36 quality of life questionnaire in surgically treated subjects were lower than the reference values, with the findings largely independent of age, curve type and severity, and the presence and magnitude of trunk deformity [33]. Adult subjects, mostly women, who had undergone a surgery in adolescence, were content with their lives but tended to start families later in life and had fewer and less satisfying sexual relationships than age- and sex-matched controls. Interestingly, in line with our findings, these outcomes were associated with the level of education [34]. Nonetheless, the findings from studies on surgically treated patients cannot be directly compared to our observations.

We observed a tendency for depressive symptoms associated with the participation in the exercise program. This finding is, on the surface, in opposition to the assumptions about the beneficial effect of exercising on mental health. Physical activity has been shown to be associated with decreased symptoms of depression and anxiety, life satisfaction, cognitive functioning, and psychological well-being. In contrast, physical inactivity is believed to be associated with the development of psychological disorders, and exercise and exercise training are considered as a treatment of depression [35, 36]. With limited evidence, people with scoliosis are encouraged to participate in physical and sports activities for the psychosocial and emotional benefits of exercising [37, 38]. However, scoliosis-specific exercise programs are not considered as physical activity due to their intentional use as a therapeutic intervention rather than leisure activity [37, 38]. Our findings also suggest that the regime of scoliosis-specific exercises is a treatment intervention, not an exercise as such. As we observed, participation in a scoliosis-specific exercise program may be associated with depressive symptoms later in life. However, our results cannot be directly balanced with any available evidence.

The multiple regression analysis revealed an adverse association of male gender with the tendency for depression (*P* < .05) and somatization (*P* = 1.87 in the logit model for ordinal variables). The latter finding corresponds with the observation from intragroup comparisons: more women, irrespective of the group, had BDI scores greater than 10 points, considered in some interpretations as a threshold of depression [23], and the only significant difference in the GHQ-28 was that of higher scores in nonscoliotic women in the “somatic symptoms” domain (Table 4). In general, rates of depression are higher and outcomes tend to be worse in women compared with men [39]. However, as regards people with scoliosis, our results can be compared only with the few available reports. Findings of a better perception of body image in scoliotic men have been reported in the Ste-Justine cohort study [12] and in a small study on braced adolescents [9]. Transient psychological effects were present during treatment in braced young adult women, and more prevalent body image disruptions in their surgically treated peers were found during the mean follow-up of seven years [10]. Nonsignificant differences between men and women fourteen years after surgery were also reported [14].

Lower level of education and being employed, unlike the family history of scoliosis, were shown to be associated with a lower tendency for depression. These observations confirm that we have to be cautious in drawing firm conclusions from this cross-sectional analysis, and that the reported associations are affected by confounding factors, which is typical for observational studies investigating real-life phenomena.

Because it was an uncontrolled observational study with a long follow-up of 16.5 years, we cautiously analyzed the obtained data with respect to limitations of the method and a number of potential confounding factors. Also, to reduce selection bias, we identified the eligible participants by applying a registry-based procedure, comprising the records of subjects from the entire local population of children and adolescents, and used a systematic procedure of random selection of the records. Participants did not differ significantly in demographic (Table 1) and clinical (Table 2) characteristics. Thus, we assume that these findings are not limited to the studied population. Nonetheless, in the sample of 144 participants, a potential selection bias resulting from refusals to participate and factors such as migration and unavailability of selected cases may limit the strength of our findings. Also, the reference values and set thresholds are lacking for both GHQ-28 and BDI, which is indicated as limitation of this instrument [17, 23]. For the availability of the Polish version, we applied the BDI-I and, accordingly, different thresholds from those proposed for the BDI-II. However, the versions are regarded as comparable, and findings from the BDI-I can be generalized to the BDI-II [24]. Finally, we had limited possibility to discuss our results in comparison with other reports. For the scarcity of outcome studies utilizing clinical measures in scoliotic patients [7], and unavailability of corresponding reports on mental health in people who participated in scoliosis-specific exercise programs—in opposition to general physical activity or exercising—we discussed studies on operated or braced patients. Moreover, other researchers typically used HRQoL rather than mental health measures. HRQoL instruments integrate the mental health domains within the general picture of a person's perceived health and well-being, with their physical and social dimensions. Screening for depression and mental health with specific instruments, such as the Beck inventory and Goldberg GHQ, is distinct from the measurements of HRQoL [40].

In conclusion, in this first outcome study of mental health of young adults with mild-to-moderate scoliosis, observed or treated with a program of scoliosis-specific exercises in adolescence, we have shown that in a long-term perspective people with mild scoliosis do not differ significantly from unaffected persons in terms of depression and other characteristics of general mental health: somatic symptoms, anxiety, insomnia, and social dysfunction. However, we found that participation in exercise treatment program in adolescence and spinal deformity even mild, or perhaps the awareness of the deformity, may have associations with depression symptoms. Thus, these findings correspond to the reports of a negative impact of scoliosis on mental health, regardless of its severity. Also, our observations may indicate that the decision to introduce a strenuous and demanding therapy should be made with caution, with proper evidence-based clinical judgment in terms of patients' needs and potential benefits, risks, and harm of an intervention.

## Figures and Tables

**Table 1:** Characteristics of the participants and intergroup differences.

Factor or domain	Total group (*n* = 144)	Unaffected (*n* = 76)	Scoliotic (*n* = 68)	*P *
Age	30.11 ± 4.11 (24–39), 30	30.11 ± 3.99 (24–38), 30	30.10 ± 4.67 (25–39), 30	.98
Women (*n*)	81	38	43	.10
Place of residence (*n*)				
Rural	8	5	3	.18
Urban ≤ 20 000	1	0	1	
Urban 20 000–50 000	2	0	2	
Urban > 50 000	133	71	62	
Marital status (*n*)				
Single	75	35	40	.17
Married/living together	69	41	28	
Education (*n*)				
Vocational	11	5	6	.88
Preuniversity/college	34	18	16	
University	99	53	46	
Intervention (*n*)				
Observation	73	41	32	.37
Exercises	71	35	36	
Scoliosis in immediate family (*n*)	48	23	25	.40

Data for age are presented as mean ± SD (range), median.

**Table 2:** Clinical characteristics of the participants with scoliosis.

Curve size (° Cobb) (*n* = 68)	Curve severity (*n, *(%))		Curve location (*n, *(%))			Scoliosis type (*n, *(%))	
	11–24° Cobb (mild)	25–40° Cobb (moderate)	Single primary Th	Single primary Th-L or L	Double major	Early-onset idiopathic*	Adolescent idiopathic**
15.16 ± 6.44 (11–36)	62 (92)	6 (8)	9 (13)	45 (66)	14 (25)	11 (16)	57 (74)

Curve size expressed as mean ± SD (range); *9 years of age; **10–16 years of age; Th: thoracic and L: lumbar.

**Table 3:** BDI and GHQ-28 scores—intergroup and intragroup comparisons.

Measures and domains	Total group (*n* = 144)	Unaffected (*n* = 76)	Scoliotic (*n* = 68)	*P *	Scoliotic		*P *
					11–24° Cobb (*n* = 62)	25–40° Cobb (*n* = 6)	
*BD *							
Total score	4.56 ± 5.31 (0–36), 3	4.68 ± 5.89 (0–36), 3	4.43/3/4.60/0–15	.77	4.45 ± 4.57 (0–13), 3	4.17 ± 5.46 (0–10), 1.5	.95
*Symptoms (n (%)) *							
Minimum	88 (61)	51 (67)	37 (54)	<.01*	33 (53)	4 (67)	<.05*
Mild	48 (33)	18 (24)	30 (44)		28 (45)	2 (33)	
Moderate	6 (4)	6 (8)	0		0	0	
Severe	2 (1)	1 (1)	1 (1)		1 (2)	0	
>10-point threshold	18 (13)	11 (14)	7 (10)	—	6 (10)	1 (17)	—
*GHQ-28 *							
A: somatic symptoms	5.36 ± 2.64 (0–13), 5	5.32 ± 2.70 (0–13), 5	5.41 ± 2.59 (0–11), 5	.82	5.48 ± 2.55 (1–11), 5	4.67 ± 3.14 (0–9), 4.5	.75
B: anxiety, insomnia	4.95 ± 3.74 (0–17), 4	4.91 ± 3.99 (0–16), 4	5.00 ± 3.46 (0–17), 5	.88	5.00 ± 3.46 (0–17), 5	5.00 ± 3.74 (0–10), 5	.99
C: functional disorders, social dysfunction	6.83 ± 1.98 (0–14), 7	6.95 ± 2.08 (0–14), 7	6.69 ± 1.87 (1–13), 7	.44	6.73 ± 1.93 (1–13), 7	6.33 ± 1.03 (5–7), 7	.67
D: depressive symptoms	1.09 ± 2.19 (0–14), 0	1.01 ± 2.14 (0–13), 0	1.18 ± 2.26 (0–14), 0	.65	1.01 ± 2.14 (0–16), 0	1.13 ± 2.33 (0–3), 0	.77
Total score (A + B + C + D)	18.01 ± 8.65 (0–56), 16	18.01 ± 9.26 (0–54), 16	18.01 ± 7.98 (0–56), 17.5	.99	18.05 ± 8.15 (4–56), 18	17.67 ± 6.62 (11–21), 16.5	.99

Data for BDI total scores and GHQ-28 are presented as mean ± SD (range), median.

BDI depressive symptoms: minimum (0–4 pts), mild (5–13 pts), moderate (14–20 pts), and severe (≥21 pts).

*Significant difference.

**Table 4:** Women versus men within the subgroups.

Measures and domains	Unaffected			Scoliotic		
	Women (*n* = 38)	Men (*n* = 38)	*P *	Women (*n* = 43)	Men (*n* = 25)	*P *
*BDI *						
Total score	4.79 ± 5.13 (0–26), 3	4.58 ± 5.89 (0–36), 3	.87	4.70 ± 5.17 (0–13), 3	3.96 ± 3.48 (0–15), 4	.52
*Symptoms (n (%)) *						
Minimum	23 (61)	28 (74)	.33	23 (53)	14 (56)	.63
Mild	11 (29)	7 (18)		19 (44)	11 (44)	
Moderate	4 (11)	2 (5)		0	0	
Severe	0	1 (3)		1 (2)	0	
>10-point threshold	7 (18)	4 (11)	—	6 (14)	1 (4)	
*GHQ-28 *						
A: somatic symptoms	5.97 ± 2.69 (2–12), 5	4.66 ± 2.58 (2–14), 4	<.05*	5.44 ± 2.75 (0–11), 5	5.36 ± 2.34 (3–10), 5	.90
B: anxiety, insomnia	5.47 ± 4.28 (0–16), 5	4.34 ± 3.66 (0–14), 4	.21	5.19 ± 3.68 (0–16), 5	4.68 ± 3.08 (0–10), 5	.56
C: functional disorders, social dysfunction	7.29 ± 2.04 (6–14), 7	6.61 ± 2.09 (6–14), 7	.15	6.67 ± 1.85 (2–13), 7	6.72 ± 1.95 (1–11), 7	.92
D: depressive symptoms	1.13 ± 2.07 (0–7), 0	.89 ± 2.23 (0–13), 0	.63	1.30 ± 2.67 (0–16), 0	.96 ± 1.31 (0–5), 0	.55
Total score (A + B + C + D)	19.53 ± 9.36 (6–37), 17	16.5 ± 9.02 (0–54) 14.5	.15	18.60 ± 8.47 (9–56), 17	17.00 ± 7.14 (9–29), 17	.42

Data for BDI total scores and GHQ-28 are presented as mean ± SD (range), median.

BDI depressive symptoms: minimum (0–4 pts), mild (5–13 pts), moderate (14–20 pts), and severe (≥21 pts).

*Significant difference.

**Table 5:** Multiple regression analysis for BDI (the zero inflation model) and GHQ-28 (the logit model for ordinal variables).

Total BDI score	Parameter estimate	Standard error	*P *	Odds ratio	95% CI
Gender (male)	−.18	.08	<.05*	.83	.71–.98
Scoliosis (<10° Cobb)	−.16	.08	<.05*	.84	.72–1.00
Intervention (observation)	.15	.08	.06	1.16	.90–1.38
Marital status (single)	−.21	.08	<.01*	.82	.68–.96
Employment (employed)	−.35	.13	<.01*	.80	.54–.92
Education (lower level)	−.35	.05	<.001*	.70	.62–.79
Scoliosis in immediate family (occurring)	−.11	.08	.15	.89	.75–1.06

GHQ-28 domain A*: somatic symptoms	Parameter value	Standard error	|*t*|	Odds ratio	95% CI

Age (older)	−.02	.03	.55	.97	.91–1.05
Gender (male)	−.56	.30	1.87	.56	.31–1.02
Scoliosis (<10° Cobb)	.06	.29	.43	1.13	.64–2.03
Intervention (observation)	.18	.30	.59	1.19	.66–2.18
Marital status (single)	.37	.30	1.21	1.45	.80–2.67
Employment (unemployed)	−.64	.45	1.33	.52	.21–1.28
Education (lower level)	−.64	.24	2.62 *P* < .05**	.52	.33–.85
Scoliosis in immediate family (occurring)	−.003	.32	.01	.99	.53–1.87

*For GHQ-28 global total and domains B, C, and D no associations were found; thus, results are not shown.

For BDI *r* ^2^was not calculated, as the zero inflated model of regression analysis was used.

For GHQ-28 McFadden pseudo *r* ^2^ = 13.65.

**Significant difference.
